# Children against antibiotics misuse and antimicrobial resistance: assessing effectiveness of storytelling and picture drawing as public engagement approaches

**DOI:** 10.12688/wellcomeopenres.16543.2

**Published:** 2021-10-22

**Authors:** Bernard Appiah, David Anum-Hagin, Martha Gyansa-Luterrodt, Elfreda Samman, Franklin Konadu Addo Agyeman, George Appiah, Gloria Odonkor, Julius Yaw Ludu, Josephine Osafo, Antonio Rene

**Affiliations:** 1 Department of Public Health, Falk College, Syracuse University, Syracuse, New York, 13244, USA; 2 Centre for Science and Health Communication, Accra, Ghana; 3 Directorate of Pharmaceutical Services, Ministry of Health of Ghana, Accra, Ghana; 4 Department of Health Promotion and Community Health Sciences, Texas A&M University School of Public Health, College Station, Texas, 77843, USA; 5 Department of Computer Science, University of Ghana, Legon, Accra, Ghana; 6 Research Program on Environment and Sustainability, Department of Environmental and Occupational Health, Texas A&M University School of Public Health, College Station, Texas, 77843, USA

**Keywords:** Antimicrobial resistance, antibiotic resistance, children, storytelling, picture drawing, public engagement

## Abstract

**Background**: Interventions delivered in schools have been found to be effective in improving knowledge of antibiotics and antimicrobial resistance (AMR) among school-aged children, particularly those in high-income countries, but the evidence is largely lacking in low- and middle-income countries. This study aimed to design, implement and assess storytelling in one school and picture drawing in another school as engagement approaches for improving knowledge, attitudes and beliefs about antibiotics and AMR among schoolchildren in Ghana.

**Methods**: Two schools with a total population of 375 schoolchildren ages 11-15 years in Tema, a city in Ghana, participated in public engagement interventions involving storytelling in one school and picture drawing in another school. The interventions included eight weeks of engagement led by science teachers and a competition held in each school. For quantitative outcome-based evaluation, schoolchildren were randomly sampled in each school (31 in the storytelling school and 32 in the picture-drawing school). Purposive sampling was also used to select 20 schoolchildren in each school for qualitative outcome-based evaluation. Respondents completed identical knowledge, attitudes and beliefs questionnaires and were interviewed at two time points (before and at most a week) after key interventions to assess changes in antibiotics and AMR knowledge, attitudes and beliefs. McNemar test was conducted to assess statistical significance between baseline and endline scores. Framework analysis was used for analysing the qualitative data.

**Results: **Picture drawing had more significant effects (both positive and negative) on schoolchildren’s AMR knowledge, attitudes and beliefs, whereas storytelling had a negative effect on children’s AMR knowledge and no significant impact on beliefs and attitudes.

**Conclusions:** Our project’s findings suggest that public engagement interventions that use picture drawing and storytelling may influence the knowledge, attitudes and beliefs of schoolchildren regarding antibiotic misuse and AMR. However, modifications are required to make them much more effective.

## Introduction

Antimicrobial resistance (AMR) represents one of the most significant challenges in health globally. According to the World Health Organization (WHO), “AMR is everywhere and has the potential to affect anyone, of any age, in any country” ^1^. AMR is associated with the inappropriate use of antibiotics and may not only contribute to increased mortality and morbidity but could lead to an increased cost for the healthcare sector ^2^.

In low- and middle-income countries (LMICs) such as those in Africa, misuse and abuse of antimicrobials have become rampant partly due to inadequate regulation of, and education about, antimicrobials, in particular antibiotics ^3– 7^. Estimates are that by 2050, there would be 10 million deaths every year globally including 4.15 million deaths and 4.73 million deaths in Africa and Asia respectively ^2^. Thus, innovative research into addressing AMR globally are urgently needed ^5^, especially with active involvement of key stakeholders such as civil society and the private sector ^6^.

Generating scientific evidence on public awareness, beliefs and attitudes regarding use of antimicrobials could help in the fight against AMR. However, communication — or public engagement — related AMR scientific evidence in Sub-Saharan Africa is rare. For example, a review of aspects of effective communication interventions to improve antibiotic use among the general public identified 14 studies, with only one having been conducted in a low- middle-income country — Thailand — and none from Africa ^8^. Similarly, a review of effectiveness of interventions to improve awareness and behaviors associated with rational use of antimicrobials among the public found 20 studies, 19 of which were conducted in high income countries, with one conducted in the LMIC of Moldova ^9^. Given the apparent lack of communication-related AMR studies aimed at the general public in Sub-Saharan Africa, the need to create and test novel, culturally appropriate strategies in educating the public on antibiotic use is fundamental in the fight against AMR in the region.

Storytelling and picture drawing could be an effective verbal and visual communication strategy for fighting AMR in Africa. Storytelling can be used as an educational tool for positive social behavioral change communication ^10^. Not only is it engaging and entertaining but it also facilitates the recollection of information ^11^. Stories can be used as a powerful health communication tool, helping people emotionally connect with health information ^12^. For example, storytelling has been used as an effective tool for health promotion including promoting colorectal cancer screening in women ^13^. A study conducted in Iran used storytelling to promote rational antibiotic use in schoolchildren using story books ^14^. After the intervention, children demonstrated a significant improvement in knowledge of antibiotic use.

Similarly, picture drawing can be used as a visual tool to provide graphic information concerning health topics. The use of picture drawing backed with a few written words can provide a more effective information or tool for health promotion than writing alone ^15^. Several studies have used visuals for educating the public on antibiotic use. For example, a study in Massachusetts, United States, aimed at improving parental antibiotic knowledge and attitudes used educational newsletters, stickers, posters, pamphlets, and fact sheets. Results demonstrated an increase in knowledge of antibiotic use ^16^. In Portugal, a study targeted at 9th graders evaluated the use of an educational slide show presentation to improve students’ knowledge on correct antibiotic use and development of antibacterial resistance. There was a significant increase in knowledge of antibiotic use after the intervention ^17^. A study conducted among children in the Czech Republic, France, and England aimed to evaluate the effectiveness of the E-bug teaching pack (a resource on microbiology and antibiotics) demonstrated improvements in knowledge of antibiotics ^18^. In Italy, a public engagement initiative for children aged 9-11 included drawing of pictures, with significant increase in knowledge of antibiotics at endline compared with baseline results ^19^.

Clearly, interventions aimed at children have an important role to play in the fight against antimicrobial resistance. This is because including children in AMR interventions not only raises awareness on antibiotic misuse in the future generation but also children are likely to share messages with their parents ^16^.

Many other AMR interventions have been aimed at schoolchildren ^20, 21^, but even so the evidence is largely lacking in Sub-Saharan Africa. There have been calls for studies from LMICs on effectiveness of communication-related AMR interventions ^8, 9^. The project aimed to create and assess storytelling and picture drawing as public engagement approaches for improving knowledge, attitudes and beliefs about antibiotics and AMR among schoolchildren in Ghana.

The design of the engagement project followed the Information-Motivation-Behavioral Skills (IMB) model. According to the IMB model, health-related behavior is dependent on whether an individual is well-informed about the behavior, is motivated to engage in the behavior, and has the adequate behavioral skills to perform the behavior ^22^. This paper describes the use and potential impact of storytelling and picture drawing as engagement approaches for improving knowledge, beliefs and attitudes about antimicrobial resistance in Ghana.

The interventions included an information component where participants were educated about safe antibiotic use, and a motivation component which included awarding of prizes to participants who took part in storytelling and picture-drawing competitions as part of the engagement strategy. Finally, changes in knowledge and behavioral skills to engage in rational antibiotic use were assessed using quantitative surveys and qualitative interviews which were conducted before and after the interventions.

## Methods

### Population/school selection

This pilot project was implemented in two Ghanaian schools at Tema Metropolis, a district in the Greater Accra region of Ghana, with a population of 292,773, in Ghana’s 2010 Population and Housing Census ^23^. Ghana’s National Drug Policy, first published in 1999, and later published in 2004 as a second edition, calls for the Ministry of Health to collaborate with other agencies including the Ministry of Education “to integrate basic information on drug use into the educational curricula” ^24^. Ghana’s National Action Plan on Antimicrobial Resistance also recognises a need to educate the public, including schoolchildren, to promote the responsible use of antimicrobials ^25^. Thus, the involvement of Ghana’s Ministry of Health and the Ghana Education Service, aimed to ensure that the findings could help provide the evidence for potential implementation of the project in schools.

The project focused on schoolchildren at the Junior High School level. In Ghana, children at this level usually are 11–15 years old. Two project team members from the Ghana Education Service selected the two schools at the Tema Metropolis, ensuring that each school had children from households considered to be from different income levels. Although different income levels were not assessed, in Ghana, children who attend public schools tend to come from households with different income levels: from the poor to the rich. Thus, private schools were avoided as they tend to have schoolchildren from relatively rich households.

### The interventions

A storytelling intervention was implemented in one school and the picture drawing intervention was implemented in another school. We selected storytelling because it is culturally appropriate in Ghana ^26^. Picture-drawing was selected because schoolchildren in Ghana already have arts lessons that include drawing pictures. Although other engagement approaches such as quiz competitions and debates are popular in Ghana, we selected storytelling and picture drawing in part because the two ideas were suggested by school children in a previous antimicrobial resistance project. Moreover, schools in Ghana currently do not have rational use of medicines as part of their curricular despite a policy recommendation ^24^. Thus, any extracurricular activities would have to be carefully implemented to not overburden children and teachers. For this reason, the study was carefully designed to ensure that only one intervention was implemented in each school. 

During the 2016/2017 academic year, when the project started, the total student population for the storytelling intervention school was 224 and that of the picture drawing school was 151.

The project was launched separately in the two schools. The science teachers (one for the storytelling intervention school and two for the picture-drawing intervention school), after being trained for three hours, received an eight-week syllabus on engaging schoolchildren with antibiotics, microbes and AMR topics ( Table 1). The training and engagement material for teachers were created by adapting relevant materials from e-Bug, free educational resource on micro-organisms and antibiotics for the lay public, with permission. Also, the teachers’ engagement materials included information from a training manual for Ghanaian civil society organizations to engage the public on AMR and a book authored by the lead investigator that has antibiotics speaking in first person narrative about correct use of antibiotics and the consequences of misuse.

**Table 1.** Classroom planned activities and educational content.

Week	Activities
1-2	For a group having 5 or six students. Have them write names of antibiotics they used before or have heard about. Ask their group leader to present Teacher ask children to have a role-play of a Ghanaian home setting to demonstrate scenarios of antibiotic misuse among parents and children
3-4	Students complete an assignment in their workbooks about the differences between viruses and bacteria, methods of transmission of resistant bacteria in communities, and the correct use of antibiotics
5-6	Teacher discusses issues of antibiotic resistance with students, including the spread of resistance through agriculture, how washing hands can help prevent the spread of bacterial infections
7-8	**Storytelling school**: Students write an essay based on the message from the animation and the common misconceptions they have learnt about during the lesson. Student read a short supplementary material provided for them in printed format from the book “ *Medicines, Using Them* *Safely*” published by the lead author. Antibiotics speak in first person in this book. In their write-ups, students discuss what the antibiotics are saying in the book. Students are asked to design the full picture by themselves as final homework in the drawing books customized for the project. **Picture drawing school:** Students draw pictures showing the misuse of antibiotics and how that could affect the health of people in the community. Ask students to read the short supplementary material provided for them in printed format on, *“Medicines, Using Them Safely.”* In their write-ups, they would discuss what the antibiotics are saying in the book. In both schools, students should consider the following points: ➢ What are the most common misconceptions around antibiotics and why might there be such widespread misunderstanding? ➢ How would tackling common misconceptions around antibiotics help to slow or prevent the rise of resistance? ➢ What methods or approaches should be used to tackle misconceptions? ➢ Personal, family or friends’ experiences of antibiotics can also be included, such as why antibiotics were taken and if the user thought they may have been unnecessary. What would have helped in this situation?

**Figure 1.**
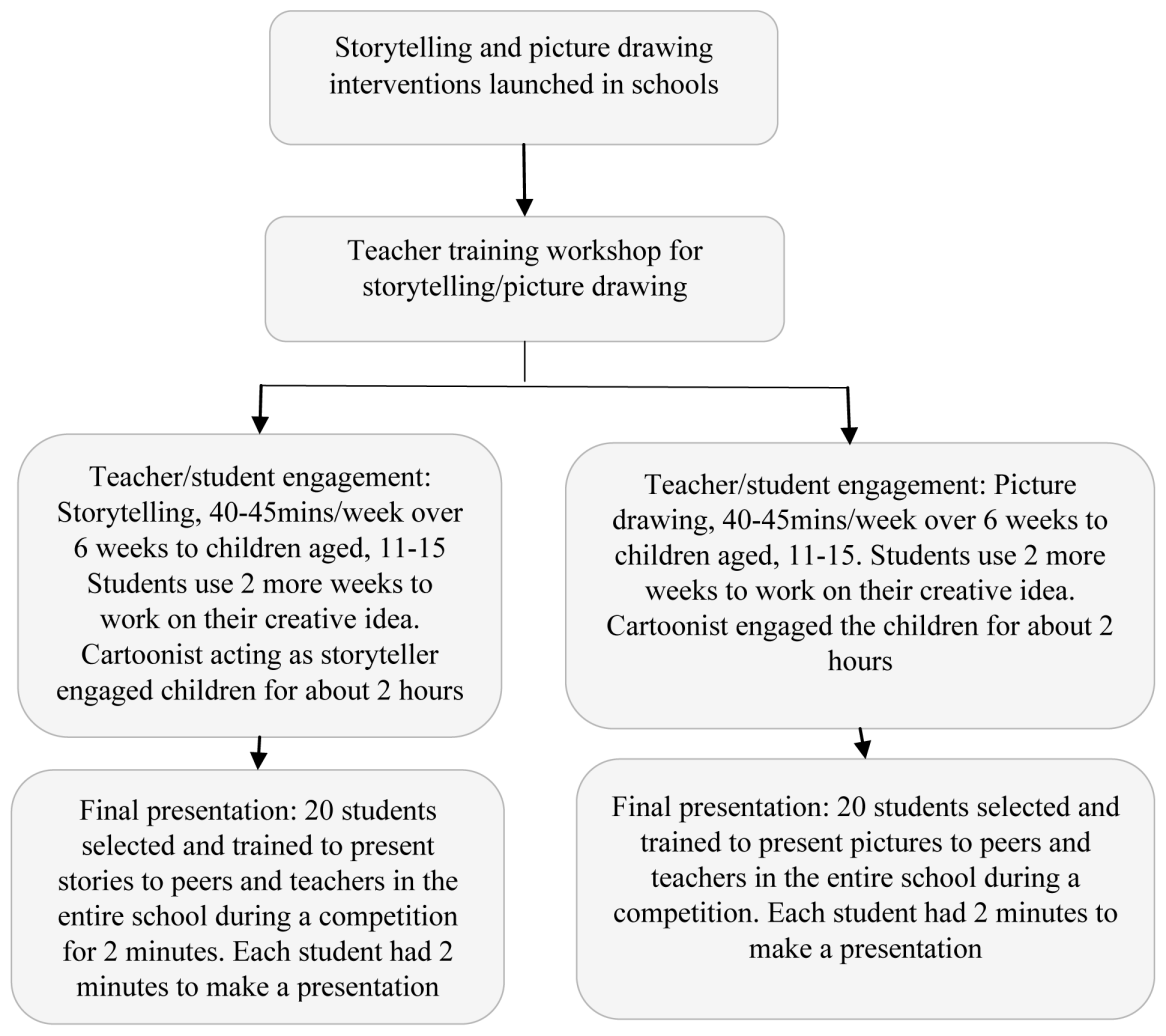
The storytelling and picture drawing interventions on antibiotics and antimicrobial resistance.

Teachers led the students on creative approaches to storytelling or picture drawing over eight weeks (September-November 2017). The top 20 stories and 20 pictures were selected by one of the co-authors and a cartoonist involved in the project based on originality, creativity, and the effectiveness of the AMR message. The selected students were further engaged to prepare for their presentations, with each student making a presentation during a competition ( Figure 1).

The top five stories and pictures received prizes (bags with science textbooks), and all the other participants received only science textbooks (see Extended data for one of the top stories and children ^27^).

### Quantitative data collection and analysis

This study used mixed methods for project evaluation. Interviewer-administered questionnaires before and after the interventions were used to evaluate the project. With the help of science teachers and the headmistress of each school, 31 students of the storytelling intervention school and 32 students of the picture-drawing intervention school were randomly selected to participate in the outcomes-based quantitative evaluation of the storytelling and picture drawing interventions respectively. In Ghana, junior high schools have three grade levels: Junior High School one (about 13 years old), Junior High School two (about 14 years old) and Junior High School three (about 15 years old). The storytelling and picture drawing intervention lessons were implemented for school children in Junior High Schools one and two. However, during the competitions, all the schoolchildren in each school had the opportunity to become participants as it was done at a time no class activity occurred. Only students in junior high school grades one and two (13–14-year olds) were randomly selected to participate in the quantitative evaluation. Students in junior high school in third grade or final year (15- year olds) were not selected to participate in the evaluation because by the time of the follow-up, they would have completed school. A trained data collector, who was not part of the implementation team, administered questionnaires to the students in order to minimize bias. The sample size was based on a single group exploratory repeated measure analysis using two repeated measures: alpha value of 0.05 and an effect size of 0.40. We selected the effect size of 0.40 as it is considered to be “large” ^28^. Using this effect size, power of 0.80 and alpha of 0.05 resulted in a sample size of 26 for each group ^28^, which was adequate for this exploratory study. However, to account for attrition, the sample size was increased to 31 in the storytelling intervention school and 32 in the picture-drawing intervention school. The survey measured changes in knowledge, attitudes and beliefs regarding antibiotic use, misuse and AMR amongst the sampled children.

The survey respondents were required to choose either “Yes,” “No” or “Don’t Know” to 12 statements related to antibiotics and AMR (see extended data ^29^). Surveys were administered at baseline and endline. A trained interviewer who was not involved in the project’s implementation was recruited to survey the children at both the pre-intervention and post-intervention stages. The endline survey occurred about one week after the competitions in each school. The questionnaire was not pilot tested because the implementing team had used most of the questions to evaluate a previous project on AMR among schoolchildren and their parents in Ghana. Prior to using the questionnaire in the previous study, pilot-testing was done, and the questions were found to be well understood by respondents. 

Based on the answers of the respondents, the letter “W” was used if the respondent answered the questions under beliefs, knowledge and attitudes wrong, and “C” if they answered the question correct. Assigning “C” as correct responses to such items has been used in a previous AMR study that used McNemar test ^30^. The quantitative data ^31^ were analysed by aggregating the proportion of respondents with correct responses to the 12 questionnaire items (see extended data ^29^) and conducting the McNemar test with RStudio v1.4 to determine statistical significance, which was set at the 0.05 level. 

### Qualitative data collection and analysis

For both the storytelling and picture drawing interventions, key informant interviews were used to explore knowledge, attitudes and beliefs regarding antibiotic use, misuse and antimicrobial resistance. Respondents were asked to give reasons for their answers (see extended data ^32^). The interviews were audio-recorded and transcribed verbatim. In each school, 20 schoolchildren took part in the outcomes-based qualitative evaluation. The children for the qualitative evaluation were purposively selected after randomly selecting their peers who were to be involved in the quantitative evaluation. Interviews were recorded and transcribed verbatim.

To analyse the qualitative data (see extended data ^33^), Google Sheet, Version 1.2 (Google LLC) was used to organize the data based on themes used in a key informant interview guide: knowledge, attitudes and beliefs. The analysis was based on framework methods ^34^. This method involved lead researcher (BA) and a coauthor (GO), who are both pharmacists, in five stages: a) becoming familiar with the interviews by reading the transcripts thoroughly, b) developing analytic framework that focuses on knowledge, beliefs and attitudes, c) indexing or identifying portions of the data that matched particular themes, d) charting or arranging specific themes, and e) mapping and interpretation. The lead researcher and a co-author resolved discrepancies through discussions. Baseline and post-intervention similarities or differences were compared for each respondent.

The reporting of the study follows STROBE guidelines ^35^.

### Ethical approval and consent

The study received ethical approval from research ethics committees of Texas A&M University (IRB2016-0656D) and the Ghana Health Service (GHS-ERC-11/07/16).

Institutional Review Board (ethical) approval was obtained from the Ghana Health Service and Texas A&M University. Written informed consent was obtained from parents of the schoolchildren, who assented to the study. Additionally, consent was obtained from headmistresses of participating schools. Respondents were also informed that participation in the evaluation was voluntary.

## Results

### Quantitative findings

At endline there were 29 schoolchildren in the storytelling intervention school and 27 schoolchildren in the picture-drawing intervention school. This represented attrition rates of two students in the storytelling intervention school and 5 students in the picture-drawing intervention school. The students who were lost to follow-up had relocated during the endline data collection.

 Most of the children in the storytelling intervention were male (69%) and almost half were 13 years old (48%). For the picture drawing intervention, most of the children were female (81%) and 13 years old (52%).

**Figure 2.**
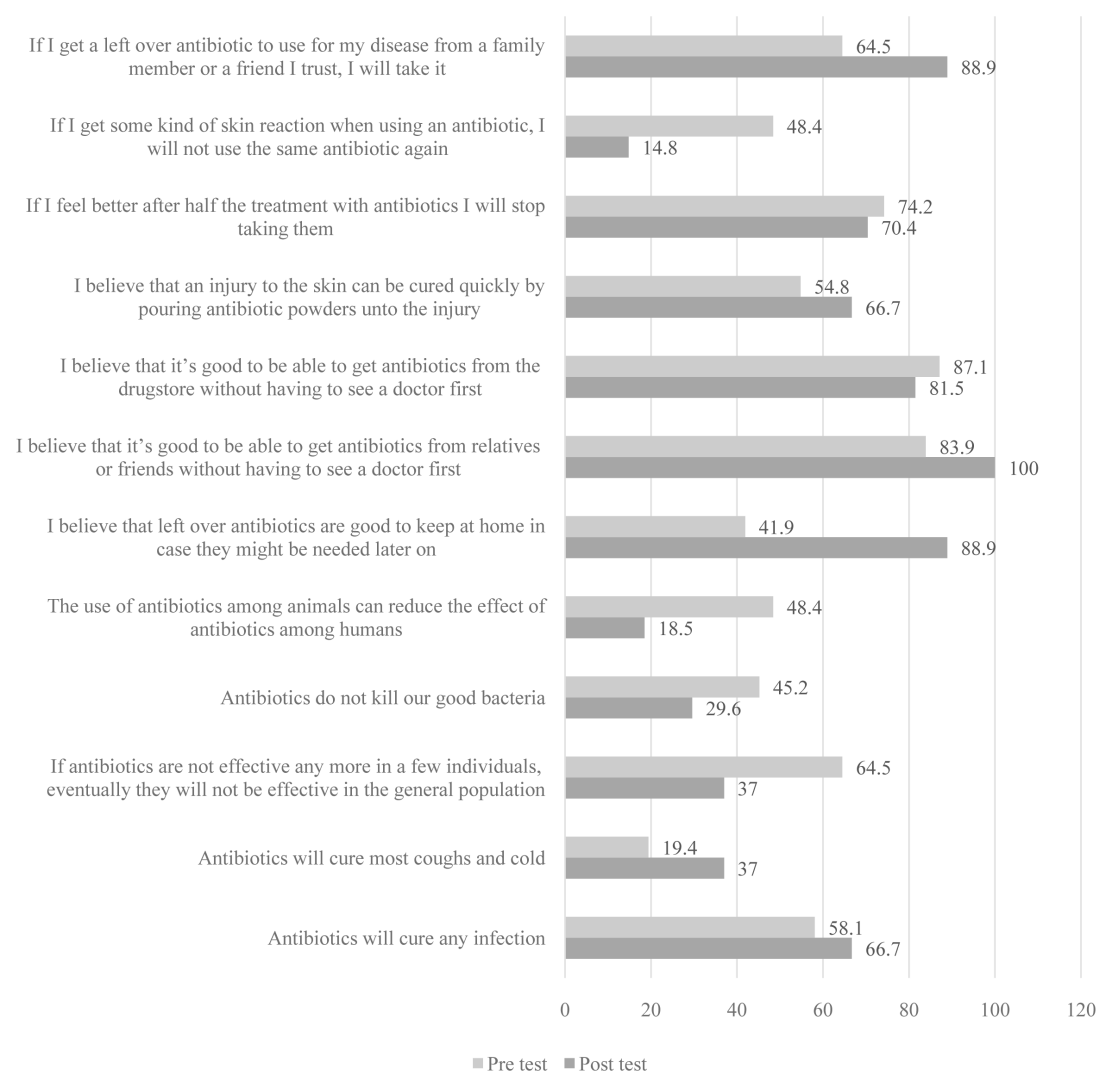
Percentage of schoolchildren who answered questions correctly before and after the picture drawing intervention.

The picture drawing intervention had six items with the proportion of correct responses at endline being more than that at baseline ( Figure 2), but only two were statistically significant as shown in Table 2.

**Table 2.** Summary of survey responses for children in picture drawing intervention.

Question	N	Pre and Post Responses ^a^				McNemar *x* ^2^	P-Value
		C&C	W&C	C&W	W&W		
**Knowledge**							
1. Antibiotics will cure any infection	27	3	6	8	10	0.071	0.789
2. Antibiotics will cure most coughs and colds	27	16	1	5	5	1.500	0.221
3. If antibiotics are not effective anymore in a few individuals, eventually they will not be effective in the general population	27	6	4	12	5	3.063	0.801
4. Antibiotics do not kill our good bacteria	27	11	8	3	5	1.455	0.228
5. The use of antibiotics among animals can reduce the effect of antibiotics among humans	27	3	2	10	12	4.083	0.043 *
**Beliefs**							
6. I believe that left-over antibiotics are good to keep at home in case they might be needed later on	27	2	-	14	11	12.071	0.001 *
7. I believe that it’s good to be able to get antibiotics from relatives or friends without having to see a doctor first	27	0	0	5	22	-	-
8. I believe that it’s good to be able to get antibiotics from the drugstore without having to see a doctor first	27	1	4	3	19	0.000	1.000
9. I believe that an injury to the skin can be cured quickly by pouring antibiotic powders onto the injury	27	5	3	6	13	0.444	0.505
**Attitudes**							
10. If I feel better after half the treatment with antibiotics, I will stop taking them	27	4	4	4	15	0.000	1.000
11. If I get some kind of skin reaction when using an antibiotic, I will not use the same antibiotic again	27	10	13	2	2	6.667	0.009 *
12. If I get a left-over antibiotic to use for my disease from a family member or friend I trust, I will take it	27	3	0	7	17	5.143	0.023 *

^a^ The pre and post responses are written via symbols between the ‘&’ where C = correct, W = wrong. For example, C&C means correct pre level and correct post level answers and W&W means Wrong pre level and wrong post level answers. *Significant at 0.05 level

The two statements were, “If I get a left-over antibiotic to use for my disease from a family member or friend I trust, I will take it” (p=0.0023, χ2 =5.143; 88.9% at endline vs 64.5% at baseline) and “I believe that leftover antibiotics are good to keep at home in case they might be needed later on” (p=0.001, χ2=12.071; 88.9% at endline vs 41.9% at baseline). The other six items that had negative effects because the proportion with correct answers at baseline was higher than that at endline also had two statements for which the results were statistically significant: “The use of antibiotics among animals can reduce the effect of antibiotics among humans” (p=0.043, χ2=4.083; 48.4% at baseline vs 18.5% at endline), and “If I get some kind of skin reaction when using an antibiotic, I will not use the same antibiotic again” (p=0.009, χ2=6.667; 48.4% vs 18.5%).

**Figure 3.**
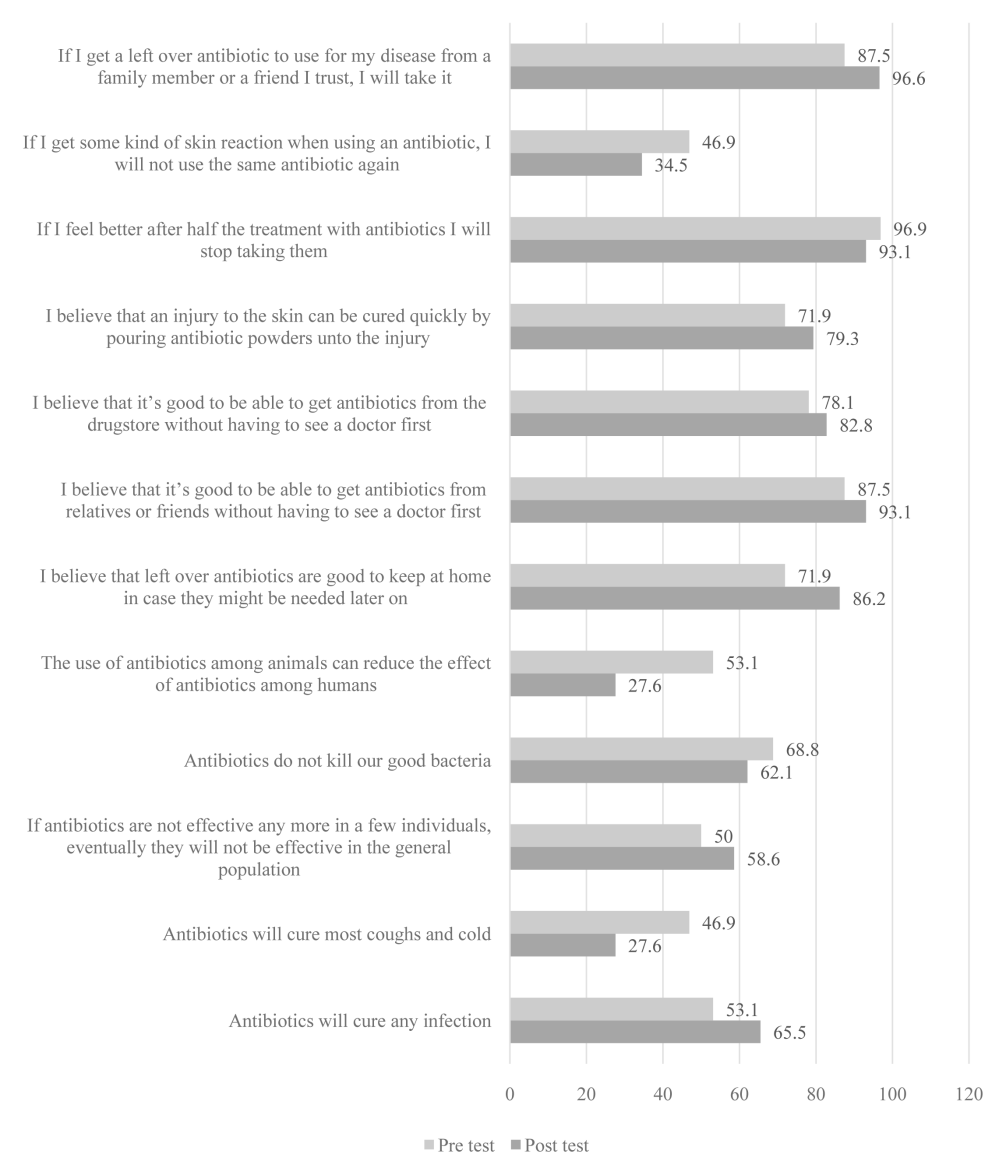
Percentage of schoolchildren who answered questions correctly before and after the storytelling intervention.

The storytelling intervention resulted in positive changes in seven items ( Figure 3) but none was statistically significant. However, of the five that showed negative effects, one was significant: “The use of antibiotics among animals can reduce the effect of antibiotics among humans” (p=0.046, χ2 =4.000 [ Table 3]; 53.1% at baseline vs 27.6% at endline.

**Table 3.** Summary of survey responses for children in storytelling intervention.

Question	N	Pre and Post Responses ^a^				McNemar *x* ^2^	P-Value
		C&C	W&C	C&W	W&W		
**Knowledge**							
1. Antibiotics will cure any infection	29	6	4	8	11	0.750	0.387
2. Antibiotics will cure most coughs and colds	29	10	11	6	2	0.942	0.332
3. If antibiotics are not effective anymore in a few individuals, eventually they will not be effective in the general population	29	10	7	6	6	0.000	1.000
4. Antibiotics do not kill our good bacteria	29	2	9	6	12	0.267	0.606
5. The use of antibiotics among animals can reduce the effect of antibiotics among humans	29	7	1	8	13	4.000	0.046 *
**Beliefs**							
6. I believe that left-over antibiotics are good to keep at home in case they might be needed later on	29	2	2	6	19	1.125	0.289
7. I believe that it’s good to be able to get antibiotics from relatives or friends without having to see a doctor first	29	1	1	3	24	0.250	0.617
8. I believe that it’s good to be able to get antibiotics from the drugstore without having to see a doctor first	29	2	3	4	20	0.000	1.000
9. I believe that an injury to the skin can be cured quickly by pouring antibiotic powders onto the injury	29	4	1	4	20	0.800	0.371
**Attitudes**							
10. If I feel better after half the treatment with antibiotics, I will stop taking them	29	0	2	1	26	0.000	1.000
11. If I get some kind of skin reaction when using an antibiotic, I will not use the same antibiotic again	29	10	9	5	5	0.643	0.423
12. If I get a left-over antibiotic to use for my disease from a family member or friend I trust, I will take it	29	0	1	4	24	0.800	0.371

^a^ The pre and post responses are written via symbols between the ‘&’ where C = correct, W = wrong. For example, C&C means correct pre level and correct post level answers and W&W means Wrong pre level and wrong post level answers. *Significant at 0.05 level

### Qualitative findings

At endline, there were 19 schoolchildren in the storytelling intervention school and 19 schoolchildren in the picture-drawing intervention school who were interviewed. The majority (53%) of the schoolchildren in the storytelling intervention were 13 years old. Likewise, for the picture drawing intervention, many (42%) of the schoolchildren were 13 years old. In general, there was some anecdotal evidence showing positive effects of the interventions in both schools as shown in Table 4.

**Table 4.** Findings from the qualitative evaluation.

Selected quotes from schoolchildren in the storytelling intervention		
Theme	Pre-test quote	Post-test quote
**Knowledge**		
What do you know about antibiotics?	“No idea”	“I know that antibiotics are drugs which have been made to reduce or kill the…to reduce the actions of micro- organisms in the body.”
**Beliefs**		
Do you believe that left over antibiotics are good to keep at home in case they may be needed later on?	“Yes. Only if they don’t expire, they will be effective”	“No because as I said they become immune to the drug the second time the disease come.”
**Attitudes**		
If you get some kind of skin reaction when using antibiotics, what will you do and why?	“I will go to the doctor because I would like to ask if it was a mistake or what I eat”	“Skin reaction? I will first of all assume maybe a chemical in the antibiotic I’m allergic to it and I will go to the doctor to prescribe another medicine for me and do some test so that I will know if there is a chemical in that medicine to which I’m allergic to.”
Selected quotes from schoolchildren in the picture drawing intervention		
Theme	Pre-test quote	Post-test quote
**Knowledge**		
What do you know about antibiotics?	“They are drugs that we take.”	“Antibiotics are medicines that are used to cure infections by bacteria.”
**Beliefs**		
Do you believe that it is good to be able to get antibiotics from relatives or friends without having to see a doctor first?	“No …because if the person has used it, it’s not for…it’s not a compound use, you have to see a doctor to give it to you not a relative or a family member.”	“No. You have to see the doctor for prescribed medicine.”
**Attitudes**		
If you feel better after half the treatment with antibiotics, what will you do and why?	“I will keep it so that whenever I get that thing again I will use it.”	“I will continue taking it. Let’s say the doctor prescribes …you take it one day and you feel better you must continue taking it.”

## Discussion

The quantitative evaluation shows that the picture drawing intervention had a statistically positive effect on one each of the attitudes and beliefs statements and a statistically negative effect on one of each of the attitudes and knowledge statements. 

The statistically positive effects on the attitudes statement “If I get a left-over antibiotic to use for my disease from a family and belief statement (“I believe that leftover antibiotics are good to keep at home in case they might be needed later on”) look promising. However, it was surprising to detect statistically negative effect on knowledge (“The use of antibiotics among animals can reduce the effect of antibiotics among humans” and (“If I get some kind of skin reaction when using an antibiotic, I will not use the same antibiotic again”). The negative effect on potential reactions on adverse events to antimicrobial likely resulted from the intervention not providing enough contexts regarding how to address adverse drug reactions. Also, the intervention did not provide more one health (human health and animal health) information.

This suggests that the picture-drawing intervention had mixed effects on knowledge on AMR and correct use of antibiotics. 

Moreover, the storytelling intervention’s statistically negative effect on the knowledge statement (“The use of antibiotics among animals can reduce the effect of antibiotics among humans”) suggests a need for future projects to better address the one health issues regarding AMR. 

While the negative result was not expected, the outcome is similar to the outcome of a community-based educational intervention on AMR in Italy ^36^. Before the Italy intervention, 47% of the surveyed individuals responded “Yes” to the statement “Antibiotics are effective against viruses but at endline the incorrect response increased to 67%, and this was statistically significant. The authors indicated: “Population knowledge and attitudes became slightly worse after the campaign in both the intervention and the control areas” ^36^. 

Despite the storytelling intervention having positive effect on one of the attitudes statements, all four statements on beliefs and two of the knowledge statements, none of these were statistically significant. This finding is similar to some results obtained in Portugal among schoolchildren aged 14–16 years old ^17^. For example, in that study the percentage of correct answers on antibiotic use increased for all five statements but only one was statistically significant: “Antibiotics can be taken at different times each day, if the daily doses are taken” (p= 0.005 in one school and p= 0.002 in another school). The negative effect identified in this current study also mirrors that of the Portugal study which found decreased effect on the statement “Antibiotics do not interact with alcohol” (94.6% at baseline and 88.9% at endline for one school and 97.3% at baseline and 95.6% and endline for another school) although neither was statistically significant. Moreover, a related study among UK schoolchildren aged 9–12 found no statistical significance in all six knowledge statements on antibiotic use ^30^.

The qualitative data provides evidence about the improved knowledge, attitudes and beliefs about antibiotic use and AMR among the children. To the best of our knowledge, our study is the first in Ghana to test the effectiveness of storytelling and picture drawing as public engagement interventions among schoolchildren in influencing knowledge, attitudes and beliefs about antibiotics and AMR. Differences between the two interventions could result from how they were implemented. The picture drawing intervention appeared to be more engaging, relying on both visual and auditory skills. Children who used this intervention used role-plays to draw the pictures and explain it orally to their teachers and students. However, the storytelling intervention was limited to writing and telling stories, which did not appear to be as engaging as that of picture drawing.

Our findings suggest that both picture drawing and storytelling may have a role in influencing the attitudes, knowledge and beliefs of children. The negative outcomes suggest that future interventions would need to pay much more attention to providing content that addresses the negative outcomes. For example, a one health approach to AMR and how to react to severe and minor side effects of antibiotics should be discussed adequately in subsequent interventions.

In general, we were not surprised that picture-drawing had significantly more effects (both positive and negative) on children than storytelling. We attribute this finding to increased engagement that occurred among children who used picture drawing intervention than their counterparts who used storytelling. For storytelling to be used as an effective engagement approach among schoolchildren to tackle antimicrobial resistance, it should be implemented as a role-play. Children are more likely to be interested in acting out a role-play rather than merely telling stories.

Our findings that the picture drawing intervention improved beliefs and attitudes about antibiotic use are consistent with that observed in previous school-based interventions in other countries including: Moldova ^21^; Czech Republic; France and England ^18^; Portugal ^17^; and the United Kingdom ^30^.

A strength of our study is that it provides the evidence for conducting quasi-randomised control trials for testing the efficacy of picture-drawing and storytelling as engagement approaches for influencing the knowledge, beliefs and attitudes about antibiotic use and antimicrobial resistance in Ghana. For example, scholars will be able to use the outcome of the picture drawing intervention to estimate the sample size and number of schools needed for conducting quasi-experimental trials on the subject in Ghanaian schools. Such trials could provide robust evidence; given that the evidence is lacking in Sub-Saharan Africa, at least based on reviews 8, 9, such trials are urgently needed. 

Another strength is that the storytelling intervention was rooted in African culture, which often relies on oratory skills. Thus, despite being less engaging than that of the picture drawing intervention, it was culturally appropriate for the setting. Both interventions can be adapted to similar settings in Ghana and other LMICs. Moreover, the use of a mixed methods approach helped provide context as found in a previous study in the UK ^37^.

Nevertheless, these findings should be interpreted with caution. The lack of data on long-term retention of knowledge, attitudes and beliefs is a limitation, thus making it difficult to assess the true success or otherwise of the interventions. We did not include control schools as was the case for the studies in Italy ^19^ and Portugal ^17^. Future interventions should consider having control schools. However, such control schools should later be exposed to the interventions if found to be effective. It is important to indicate that the two interventions required time commitments from science teachers. Given that antibiotic use is not a lesson for junior high schools in Ghana, finding extra time to implement interventions is key. Researchers and practitioners eager to engage schoolchildren with antibiotics and AMR messages may need to be sensitive to the needs of schoolchildren and science teachers. The possibility of schoolchildren providing socially acceptable answers may also be a limitation of the study. Also, only two schools were used for the study, thus limiting the generalizability of the study findings in Ghana.

## Conclusion

Although the actual behaviour of the schoolchildren was not assessed in terms of antibiotic use, the current study provides the initial evidence in Ghana to suggest that picture drawing has significantly positive effects on beliefs and attitudes, and negative effect on attitudes and knowledge of schoolchildren on antibiotics and AMR. Also, storytelling had a significantly negative effect on influencing the knowledge of schoolchildren regarding how agriculture could contribute to AMR. Both interventions need to be modified to positively influence the knowledge, attitudes and beliefs of school-aged children in regard to antibiotics use and antimicrobial resistance.

## Data availability statement

### Underlying data

Harvard Dataverse: Data on antimicrobial resistance storytelling and picture drawing engagement interventions among schoolchildren in Ghana, https://doi.org/10.7910/DVN/G0VOCP ^31^

This project contains the following underlying data:

- Data on storytelling intervention
- Data on picture drawing intervention

Data are available under the terms of the Creative Commons Zero "No rights reserved" data waiver (CC0 1.0 Public domain dedication).

Figshare: Qualitative Data. Storytelling and picture drawing as children's public engagement approaches for addressing antimicrobial resistance in Ghana, https://doi.org/10.6084/m9.figshare.14206757.v2 ^33^

This project contains the following underlying data

- Qualitative data on picture drawing and storytelling interventions

Data are available under the terms of the Creative Commons Attribution 4.0 International license (CC-BY 4.0).

### Extended data

Figshare: Selected story and picture drawing on antimicrobial resistance from children project in Ghana. http://www.doi.org/10.6084/m9.figshare.16757467 ^27^

Figshare: Correct answers to quantitative questions, https://doi.org/10.6084/m9.figshare.14207006 ^29^

Figshare: Interview guide, https://doi.org/10.6084/m9.figshare.14206967 ^32^ Data are available under the terms of the Creative Commons Attribution 4.0 International license (CC-BY 4.0).

### Reporting guidelines

Figshare: STROBE checklist for ‘Storytelling and picture drawing as children's public engagement approaches for addressing antimicrobial resistance in Ghana’, https://doi.org/10.6084/m9.figshare.14207099 ^35^

Data are available under the terms of the Creative Commons Attribution 4.0 International license (CC-BY 4.0).
