# Supplementary material for: Children against antibiotics misuse and antimicrobial resistance: assessing effectiveness of storytelling and picture drawing as public engagement approaches
Source: Wellcome Open Res. 2021 Oct 22;6:202. Originally published 2021 Aug 9. [Version 2] doi: 10.12688/wellcomeopenres.16543.2 (PMC8546736; doi:10.12688/wellcomeopenres.16543.2)
Supplement: Peer review history

# Peer review: Reviewer response for version 2

**DOI:** 10.21956/wellcomeopenres.19109.r46587

**Published:** 2021-10-25

**Authors:** Renly Lim

**Affiliations:** 1 Clinical and Health Sciences, University of South Australia, Adelaide, SA, Australia

Thank you for addressing all suggestions adequately. I have no further comments.

Is the work clearly and accurately presented and does it cite the current literature?

Partly

If applicable, is the statistical analysis and its interpretation appropriate?

Partly

Are all the source data underlying the results available to ensure full reproducibility?

Yes

Is the study design appropriate and is the work technically sound?

Partly

Are the conclusions drawn adequately supported by the results?

No

Are sufficient details of methods and analysis provided to allow replication by others?

No

Reviewer Expertise:

Medication safety, quality use of medicine, community engagement

I confirm that I have read this submission and believe that I have an appropriate level of expertise to confirm that it is of an acceptable scientific standard.

# Peer review: Reviewer response for version 1

**DOI:** 10.21956/wellcomeopenres.18229.r45391

**Published:** 2021-08-23

**Authors:** Dorcas Kamuya

**Affiliations:** 1 Health Systems and Research Ethics, KEMRI-Wellcome Trust Research Programme, Kilifi, Kenya

Given the paucity of literature from SSA on effective approaches to change attitude, knowledge and perceptions of antibiotic misuse, the paper is timely. Also, it is commendable that the authors are brave and have been open to publishing a paper showing results that were not what was intended. In this way, it helps other researchers to learn. Some areas that could strengthen the manuscript:

1. The qualitative data is under-represented in the manuscript and could have provided insights into the results 
2. It would be very helpful if a sample of the stories and the pictures are presented - as could help the reader understand why the approach seems to have had a negative effect on some key questions.
3. The sample size of 31 and 32 for the two arms seems to be small, were there assumptions made that could have contributed to this (e.g. how was the effect size of 0.401 arrived at)?
4.  Under discussion, while the authors have discussed the results, they could also address why the outcomes were the way they were - what else could have contributed to the outcome? 
5. Were there further actions/activity taken to try to correct some of the 'mis'-perceptions that the results showed with antibiotic use? It would be helpful if the authors would indicate how this was handled.

Is the work clearly and accurately presented and does it cite the current literature?

Yes

If applicable, is the statistical analysis and its interpretation appropriate?

Yes

Are all the source data underlying the results available to ensure full reproducibility?

Partly

Is the study design appropriate and is the work technically sound?

Yes

Are the conclusions drawn adequately supported by the results?

Yes

Are sufficient details of methods and analysis provided to allow replication by others?

Yes

Reviewer Expertise:

Engagement and ethics

I confirm that I have read this submission and believe that I have an appropriate level of expertise to confirm that it is of an acceptable scientific standard.

# Peer review: Reviewer response for version 1

**DOI:** 10.21956/wellcomeopenres.18229.r45390

**Published:** 2021-08-18

**Authors:** Renly Lim

**Affiliations:** 1 Clinical and Health Sciences, University of South Australia, Adelaide, SA, Australia

I believe that the findings of the project will add valuable information to the existing literature; however, I have some major concerns around the study methodology and interpretation of results (and some minor comments) as listed below.

 It will be helpful to the readers if the authors can include more details in the abstract e.g. how many students completed the questionnaires, how many people were interviewed, what tests were used (e.g. the authors mentioned “more significant effects”) and methods used to analyse the qualitative interviews.

 It was not clear to me initially, when reading the title and background that the storytelling and picture drawing were implemented separately in different schools. I was under the impression that the engagement activities consisted of both storytelling and picture drawing. I would encourage the authors to make this clearer in the title and background. I wonder why the authors decided to implement only one intervention in each school (assuming the authors think one intervention might be more effective than the other) but did not compare then how compare the effectiveness of the two interventions.

 Also, why did the authors choose storytelling and picture drawing? What are the other engagement activities that are popular in schools in Ghana?

 The authors stated that the questionnaire was not pilot tested because the questions were used to evaluate a related project. Was there any pre-testing or validation done with the questions prior to use in the other project? Looking at the questions included in the quantitative evaluation, the questions all seem to be testing knowledge of the students and were assigned a “correct response”. There should not be a “correct” response if the authors were trying to understand beliefs or attitudes of the students.

 Did all the students in both schools receive the intervention, and of these, only some of the students were selected for quantitative evaluation? This is not clear as the results mentioned only 29 and 27 schoolchildren in each of the groups.

 Looking at the results, it seems odd to me that there was a negative effect on the schoolchildren’s knowledge on AMR. Surely, if the children knew that “If antibiotics are not effective anymore in a few individuals, eventually they will not be effective in the general population” prior to the intervention, they would also know this statement is true after the intervention?

 It isn’t clear to me how the authors did the interview and analysed the data. The results presented in Table 4 looked like the students were asked to answer three pre-defined questions on knowledge, beliefs and attitudes, instead of the authors conducting qualitative interviews.

 The authors concluded that the public engagement interventions using picture drawing and storytelling may influence knowledge, attitudes and beliefs of schoolchildren. However, looking at the results, the interventions seem to have variable and negative effect on knowledge. What could have contributed to this finding? Is this truly the result of the intervention or could this be simply due to chance?

Is the work clearly and accurately presented and does it cite the current literature?

Partly

If applicable, is the statistical analysis and its interpretation appropriate?

Partly

Are all the source data underlying the results available to ensure full reproducibility?

Yes

Is the study design appropriate and is the work technically sound?

Partly

Are the conclusions drawn adequately supported by the results?

No

Are sufficient details of methods and analysis provided to allow replication by others?

No

Reviewer Expertise:

Medication safety, quality use of medicine, community engagement

I confirm that I have read this submission and believe that I have an appropriate level of expertise to state that I do not consider it to be of an acceptable scientific standard, for reasons outlined above.
